# EBUS-TBNA Cytological Samples for Comprehensive Molecular Testing in Non–Small Cell Lung Cancer

**DOI:** 10.3390/cancers13092084

**Published:** 2021-04-25

**Authors:** Roberto Martin-Deleon, Cristina Teixido, Carmen Mª Lucena, Daniel Martinez, Ainhoa Fontana, Roxana Reyes, Mireia García, Nuria Viñolas, Ivan Vollmer, Marcelo Sanchez, Pedro Jares, Francisco Manuel Pérez, Naiara Vega, Elba Marin, Ramón Mª Marrades, Carlos Agustí, Noemi Reguart

**Affiliations:** 1 Department of Respiratory Medicine, Thoracic Oncology Unit, Hospital Clínic of Barcelona, 08036 Barcelona, Spain; robermartin48@gmail.com (R.M.-D.); cmlucena@clinic.cat (C.M.L.); afontana@clinic.cat (A.F.); marrades@clinic.cat (R.M.M.); cagusti@clinic.cat (C.A.); 2 Translational Genomic and Targeted Therapeutics in Solid Tumors, August Pi i Sunyer Biomedical Research Institute (IDIBAPS), 08036 Barcelona, Spain; teixido@clinic.cat (C.T.); rmreyes@clinic.cat (R.R.); nvinolas@clinic.cat (N.V.); elmarin@clinic.cat (E.M.); 3 Department of Pathology, Thoracic Oncology Unit, Hospital Clínic of Barcelona, 08036 Barcelona, Spain; dmartin1@clinic.cat (D.M.); garcia01@clinic.cat (M.G.); pjares@clinic.cat (P.J.); fmperez@clinic.cat (F.M.P.); NVEGA@clinic.cat (N.V.); 4 Department of Medical Oncology, Thoracic Oncology Unit, Hospital Clínic of Barcelona, 08036 Barcelona, Spain; 5 Department of Radiology, Thoracic Oncology Unit, Hospital Clínic of Barcelona, 08036 Barcelona, Spain; vollmer@clinic.cat (I.V.); msanche@clinic.cat (M.S.)

**Keywords:** EBUS, cytology, NSCLC, PD-L1, nCounter, NGS

## Abstract

**Simple Summary:**

Endobronchial-ultrasound transbronchial needle aspiration (EBUS-TBNA) is essential in the diagnosis and staging of NSCLC, but its usefulness for a full molecular characterization remains controversial. The aim of this prospective study was to assess if EBUS-TBNA samples were reliable for a comprehensive molecular and immunohistochemical testing in NSCLC. We prospectively evaluated EBUS-TBNA specimens for molecular characterization showing that they are useful for NSCLC genotyping and have the same potential to improve the selection of patients for personalized therapies as bronchial biopsy samples. EBUS-TBNA samples are reliable samples for NSCLC genotyping with the consequent potential to improve patient’s selection for targeted therapies.

**Abstract:**

Clinical guidelines promote the identification of several targetable biomarkers to drive treatment decisions in advanced non-small cell lung cancer (NSCLC), but half of all patients do not have a viable biopsy. Specimens from endobronchial-ultrasound transbronchial needle aspiration (EBUS-TBNA) are an alternative source of material for the initial diagnosis of NSCLC, however their usefulness for a complete molecular characterization remains controversial. EBUS-TBNA samples were prospectively tested for several biomarkers by next-generation sequencing (NGS), nCounter, and immunohistochemistry (PD-L1). The primary objectives were to assess the sensitivity of EBUS-TBNA samples for a comprehensive molecular characterization and to compare its performance to the reference standard of biopsy samples. Seventy-two EBUS-TBNA procedures were performed, and 42 NSCLC patients were diagnosed. Among all cytological samples, 92.9% were successfully genotyped by NGS, 95.2% by nCounter, and 100% by immunohistochemistry. There were 29 paired biopsy samples; 79.3% samples had enough tumor material for genomic genotyping, and 96.6% for PD-L1 immunohistochemistry. A good concordance was found between both sources of material: 88.9% for PD-L1, 100% for NGS and nCounter. EBUS-TBNA is a feasible alternative source of material for NSCLC genotyping and allows the identification of patient candidates for personalized therapies with high concordance when compared with biopsy.

## 1. Introduction

Lung cancer is the most common malignancy worldwide and remains the first cause of cancer death in both men and women. Non-small cell lung cancer (NSCLC) constitutes 85% of all lung cancers and 60% of them are diagnosed in advanced stages with a median five-year survival of 15% [1]. However, the use of predictive cancer biomarkers in advanced NSCLC for specific targeted therapies and immunotherapies has emerged in recent decades, increasing positive patient outcomes [2]. Genomic profiling is now a standard of care in the routine diagnostic workup of patients with advanced lung cancer and a necessity to drive treatment decisions in clinical practice [3]. Testing of *EGFR*, *BRAF*, *ALK*, *ROS1*, and PD-L1 expression [2,4,5,6,7] is considered mandatory in patients with advanced disease and the most recently updated guidelines encourage the evaluation for several other evolving targets such as *NTRK*, *RET, MET* exon14 skipping (*MET*∆ex14), *HER2*, and *KRAS* [2]. With the growing demand for gene testing in lung cancer, multiplex approaches are increasingly necessary to allow the study of several genes at the same time [5,8].

Bronchoscopy has been traditionally employed in the diagnosis of lung cancer. The recent implementation of endobronchial ultrasound-guided transbronchial needle aspiration (EBUS-TBNA) as an additional tool in the endoscopic exploration allows us not only to obtain a specific histological diagnosis but also to establish an accurate nodal mediastinal staging in a unique investigation. This results in a reduction in the time-to treatment decision [9], which is of paramount importance since timeliness of lung cancer care is a fundamental quality indicator.

Although biopsies are still considered the gold standard source of material for genetic testing [10,11,12,13], current molecular testing guidelines highlight that any source of material, including cytology samples, with adequate tumor cellularity may be used for diagnosis or therapy-predictive biomarker testing [5,14]. This is of relevance as small biopsy and cytology samples may represent the sole diagnostic material for diagnosis in up to two thirds of patients [15,16], and formalin-fixed paraffin-embedded (FFPE) biopsies have frequently limited tumor content, since many diagnostic tests are required [17].

This situation prompted us to investigate the yield of EBUS-TBNA specimens, a cytological methodology, using a comprehensive multiplex genotyping based on combined next-generation sequencing (NGS) (DNA) and nCounter (RNA) multiplex testing [18], as well as PD-L1, in a series of patients with newly diagnosed NSCLC at our institution. We also aimed to assess the reproducibility of results between cytological specimens and paired tissue biopsies taken from the same tumor.

## 2. Materials and Methods

### 2.1. Patients

Patients from Hospital Clinic Barcelona (Barcelona, Spain) with suspected lung cancer were evaluated by the Respiratory Department where the different diagnostic and staging procedures were indicated and performed according to international recommendations [9]. All cases were discussed in multi-disciplinary team meetings composed of various professionals involved in the care of lung cancer patients. Patients undergoing an EBUS-TBNA with confirmed diagnosis of stage III or IV NSCLC were prospectively included in the study. Patients were staged according to the International Association for the Study of Lung Cancer classification (eighth edition) [19]. Patients who were diagnosed on cytology were not required to have a confirmation on biopsy.

### 2.2. EBUS-TBNA

EBUS-TBNA was performed in the Bronchoscopy Unit on an outpatient basis using a convex probe ultrasound bronchoscope (EB-1970UK 2.0, Pentax ^®^, Pentax Medical, New Jersey, USA). Conscious sedation was performed by an anesthesiologist using a continuous infusion of propofol and remifentanil. The bronchoscope was inserted orally and a systematic echographic nodal evaluation was done according to clinical guidelines [2], starting with the exploration of N3 hilar and interlobar stations then mediastinal N3 stations, and finally N2 stations. Once the suspected lymph node was ecographically located, its minimum diameter was measure and a dedicated 22-gauge needle (ECHO-HD-22-EBUS-P, Echotip ^®^ ultra, Cook Medical, Bloomington, USA) was used to obtain cytological samples. An expert cytopathologist carried out a rapid on-site evaluation (ROSE) with diff-quik staining for the assessment of the amount and viability of the tumor cells. Based on this, a number of passes were made, ranging from 1 to 13, with smears or blocks being carried out as appropriate. A lymph node was considered to be non-malignant when at least three needle passes evidenced normal lymphocytes with no atypical cells.

Cytology smears were suitable for molecular testing when at least 300 tumor cells were observed. Smears with an insufficient tumor cell percentage (<20%) were considered not evaluable in order to avoid false negative results. In those cases, for which low-tumor cellularity cytology smears were observed, cells collected from up to three smears were combined for DNA and/or RNA isolation. Cell blocks were prepared from normal saline with Histogel ^®^ (Thermo Fisher Scientific, Waltham, USA).

### 2.3. Flexible Bronchoscopy

Patients with suspicion of bronchial infiltration of the primary tumor underwent, after the EBUS-TBNA procedure, a flexible bronchoscopy (FB) for obtaining biopsy samples. A total of 3 to 6 bronchial biopsies were obtained with a single-use biopsy forceps (Radial Jaw ™ 4, Boston Scientific ^®^, Marlborough, USA) that were preserved in 4% “ready to use” formol (Histofix ^®^, Panreac Quimica, Castellar del Vallès, Spain) for further histological and molecular analysis.

### 2.4. Genetic Testing

After diagnosis, biopsy (when feasible) and/or EBUS-TBNA cytology material was used to perform the molecular analyses. The best scenario was to have enough tumor cells to perform the three different techniques: NGS (DNA mutations), nCounter (RNA gene fusions and *MET*∆ex14), and immunohistochemistry (IHC) for PD-L1 expression (see supplementary material and methods). For NGS and nCounter only smears were used, while for PD-L1, smears or cell-blocks were used according to availability. All immunohistochemical studies were evaluated by two anatomical pathologists specialists (CT and DM). For controversial cases, a consensus was reached over a double headed microscope. References [18,20,21,22,23] are cited in the supplementary material.

### 2.5. Statistical Analysis

Descriptive statistics were tabulated and presented, including mean, standard deviation, median, minimum value and maximum value, and range for continuous variables, or percentages and frequencies for categorical variables. Characteristics of all patients included in the study were compared with Student’s t-test for mean values between groups. The Kappa value was used to evaluate concordance between measurements. For the statistical analysis, the SPSS program version 24.0 (SPSS, Chicago, IL, USA) was used. All tests were performed at a significance level of *p* = 0.05 and calculated at confidence level 1 − α = 0.95. Cases with missing data for analyzes were omitted and the remaining data was analyzed.

## 3. Results

### 3.1. Patients

**Figure 1:**
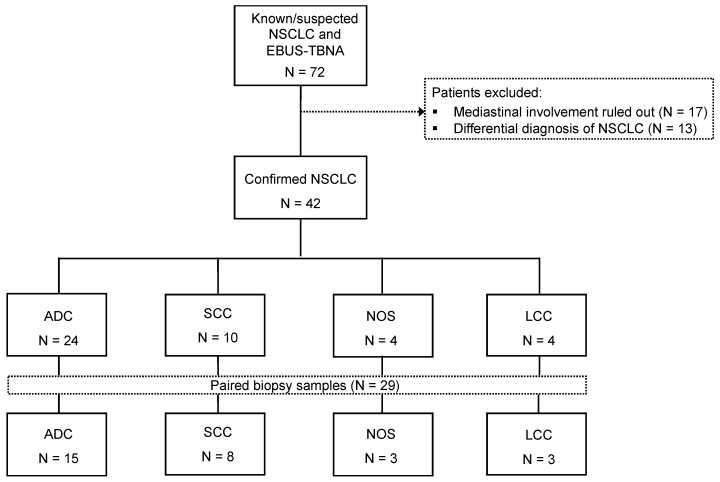
Flow chart of the patient cohort included in this study. Abbreviations: ADC, adenocarcinoma; LCC, large cell carcinoma; NOS, not otherwise specified; SCC, squamous cell carcinoma; NSCLC, non-small cell lung cancer.

During the study period (August 2018 to December 2019), 72 patients with suspected stage III or IV lung cancer underwent EBUS-TBNA for mediastinal evaluation. In 42 out of these 72 patients, metastatic mediastinal lymph nodes from NSCLC were confirmed (58.3%). The presence of malignant cells was ruled out in 17 cases (23.6%), whereas an alternate diagnosis was provided in 13 cases (18.1%), including nine patients with small-cell lung cancer, one with breast neoplasia, one with biliopancreatic cancer, one with urothelial carcinoma, and one case of pulmonary tuberculosis (Figure 1).

The final study population consisted of 42 patients with NSCLC, of whom 31 (73.8%) were men and 11 (26.2%) were women (Table 1). The mean age was 67.1 years (range 52–83). The majority of patients were smokers (47.6%; 20/42). A total of 57.1% had extensive or stage IV disease (24/42) and 42.9% locally-advanced disease (18/42). Adenocarcinoma was the most common histological subtype (57.2%; 24/42), followed by squamous cell carcinoma (23.8%; 10/42), non-otherwise specified (NOS) and large cell carcinoma (9.5%; 4/42, each) (Table 1).

### 3.2. Flexible Bronchoscopy and EBUS-TBNA Representativeness

A total of 78 different anatomical regions (according to the lymph node map of the International Association for the Study of Lung Cancer (IASLC)) were sampled with EBUS-TBNA, 50 (64.1%) on mediastinum, 13 (16.7%) on lobar nodes. In 15 patients, cytology samples were obtained directly from the peribronchial primary tumor mass (Table 2). The most commonly sampled nodal locations were subcarinal (28.2%; 22/78), followed by right lower paratracheal (24.3%, 19/78). No clinically significant complications appeared in any patient during the procedure, but in four the following two weeks (2 hemoptysis and 2 respiratory infections).

Sixty-eight out of the 78 anatomical regions were positive (87.2%), 9 negative (11.5%), and one not evaluable (1.3%) due to the absence of a confirmatory lymphocytic background. The average lymph node size on EBUS was 13.7 mm, the mean standard uptake value (SUV) was 16.9, and the mean number of punctures per adenopathy was 6. Positive node regions were significantly larger (14.5 vs. 8.8 mm, *p* = 0.029) and had an increased SUV uptake (SUV max mean difference 19.3 vs. 0.0, *p* = 0.002) compared to the negative nodes. Furthermore, FB exploration showed macroscopic evidence of bronchial infiltration by the tumor in 18 of the 42 patients (42.9%).

### 3.3. Molecular Analysis of Cytological Material Obtained by EBUS-TBNA

Overall, 39 out of 42 (92.8%) patients samples yielded sufficient material to successfully proceed with all genomic tests (92.8% (39/42) for NGS, 95.2% (40/42) for nCounter and 100% (42/42) for PD-L1). All three cases with insufficient material corresponded to multiplexed testing, whereas all samples were sufficient for PD-L1 IHC testing (Supplementary Figure S1). There were two samples with a non-evaluable result, accounting for one Oncomine Solid Tumor (OST) NGS and another PD-L1 IHC due to low quality of the DNA and insufficient number of tumor cells, respectively.

**Figure 2:**
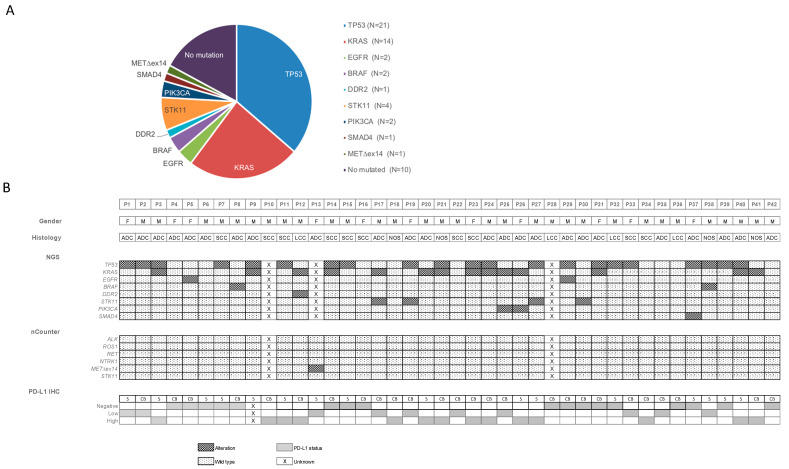
Molecular characterization of EBUS-TBNA samples. (**A**) Pie chart representing frequencies (%) of the examined genes identified from patients successfully genotyped by next-generation sequencing. Frequencies are expressed as the percentage of positive samples for each molecular alteration relative to the total number of patients with an informative molecular result. (**B**) Heatmap displaying the sex, NSCLC histological subtype and molecular characteristics of the 42 patients included in the study. Abbreviations: ADC, adenocarcinoma; CB, cell block; F, female; IHC, immunohistochemistry; LCC, large cell carcinoma; M, male; NGS, next-generation sequencing; P, patient; SCC, squamous cell carcinoma; S, smear.

Forty-seven somatic alterations were identified in 29 cases, representing 74.4% of the samples successfully tested with OST NGS (29/39). The genes most commonly detected were *TP53* (53.8%, 21/39), *KRAS* (35.9%, 14/39), and *STK11* (10.3%, 4/39). Other genes identified with lower incidence were *EGFR* (5.1%, 2/39), *BRAF* V600E (5.1%, 2/39), *PIK3CA* (5.1%, 2/39), *DDR2* (2.6%, 1/39) and *SMAD4* (2.6%, 1/39) (Figure 2). Molecular alterations were more commonly found in women than men (80%, 8/10 and 72.4%, 21/29 respectively), whereas cases with co-mutations per sample were very-similar between sexes (men with 50%, 21/42 vs. women with 53.3%, 8/15).

The customized lung cancer nCounter panel (RNA test) was successfully performed on 40 samples (95.2%), all of them providing an evaluable result (Figure 2). All genes included in the panel (*ALK, ROS1, RET*) were negative, except for a *METex∆14* that resulted positive in one sample (2.5%; 1/40).

**Figure 3:**
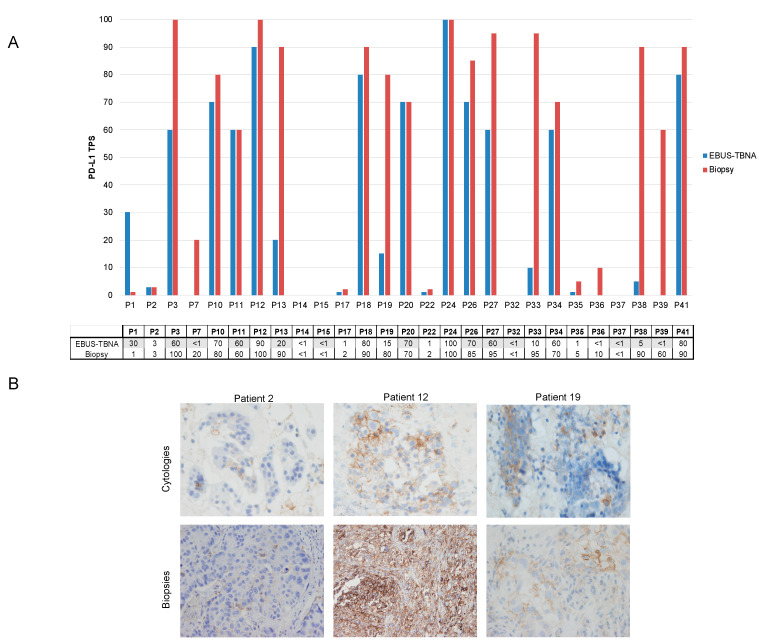
PD-L1 immunostaining in matched cytology and biopsy samples. (**A**) Bar graphs showing the comparative values of PD-L1 TPS values between the EBUS-TBNA and biopsy samples (N = 27). Each bar chart represents one sample, with the EBUS-TBNA specimen in the blue bar and paired excision in the red bar. EBUS-TBNA TPS PD-L1 results from smear samples are colored grey to differentiate them from FFPE cell blocks. (**B**) Representative images of two patients with concordant PD-L1 expression between samples (patients 2 and 12) and one patient with discordant results (patient 19). Patient 2 presented low PD-L1 expression in matched samples, while patient 12 expressed high levels of PD-L1 in both samples. In patient 19, a low positivity was observed in the cell block, while high expression levels were seen in the paired biopsy specimen. Magnification × 400. Abbreviations: EBUS-TBNA, endobronchial ultrasound-guided transbronchial needle aspiration; P, patient; TPS, tumor proportion score.

PD-L1 expression was successfully evaluated in all samples (100%, 42/42). Among them, 16 (38.1%) were smears and 26 (61.9%) cell blocks. Cases were classified according to the Tumor Proportion Score (TPS) and both types of samples (smear and cell block) contained cases within the three PD-L1 categories. PD-L1 was negative in 17 patients (40.5%; 17/42), low-positive in ten (23.8%; 10/42), and high-positive in 14 patients (33.3%; 14/42) (Figure 3).

### 3.4. Comparison of Paired EBUS-TBNA Cytology and Biopsy Samples by Targeted NGS and NCounter

Overall, FFPE samples yielded sufficient material to successfully proceed with all study testing (OST NGS, nCounter, and PD-L1 IHC) in 22 out of 29 patients (75.8%). There were six cases with insufficient material to proceed with all or any of the multiplex testing, and one sample was also insufficient for PD-L1 IHC testing. Overall, results for NGS and nCounter using biopsies were provided in 79.3% (23/29) of samples (Supplementary Figure S1). In two cases (patients 1 and 13), there was not enough tumor cell content for both DNA and RNA analyses. In patient 1, NGS was prioritized over the nCounter technique because no targetable alteration was found in the cytology material, whereas for patient 13, nCounter technique was prioritized because a *MET*∆ex14 was identified from the EBUS-TBNA material.

Twenty-nine cases had a paired cytology/FFPE biopsy for comparison purposes (Figure 1). Table 3 shows the concordance of the NGS results between the cytology and the biopsy samples. Among the 23 samples with an NGS result and paired cytology/FFPE biopsy, 18 cases were positive for any alteration. There was full agreement in the positive and negative results between both sample sources. Most of the reported results were the same (91.3%, 21/23) and only two samples (patients 9 and 37) showed discrepancies regarding co-mutations. In the first case, both cytology/FFPE biopsy agreed with a common *KRAS* mutation but co-mutations detected were different, with *TP53* in the cytology and *ALK*/*FGFR2* point-mutations in the biopsy. In the second sample, cytology resulted in a *TP53* and *SMAD4* mutation, whereas only *TP53* alteration was identified in the biopsy. The number of agreements expected by chance was 15.2 (66% of the observations).

For the nCounter technique, all of the results were concordant (23/23, 100%) with the number of agreements expected by chance 21.1 (91.7% of the observations). As mentioned above, only one patient resulted positive for the *MET*∆ex14 mutation in both cytology and biopsy.

### 3.5. PD-L1 Expression in Paired Cytology and Biopsy Samples from Patients with Advanced NSCLC

The PD-L1 status was successfully evaluated by IHC in 96.5% (28/29) of the biopsies (Supplementary Figure S1). Among them, PD-L1 testing was positive in 24 out of 28 (85.7%), including low-positive in seven patients (28.6%; 8/28) and high-positive in 16 patients (57.1%; 16/28).

A total of 27 paired cases were available for PD-L1 expression comparison between paired cytology/FFPE biopsy, as one EBUS specimen was not evaluable. When comparing the PD-L1 classification in three categories (negative, low-, and high-positive), we obtained a moderate correlation (Table 4). The kappa value was 0.59 (95% CI 0.344 to 0.838), standard error of kappa 0.126. Seven tumors were discordant, with an overall agreement of 74.1% (Figure 3A). High-positive PD-L1 TPS were more frequently observed in the biopsies than the cytologies (16 vs. 11 cases, respectively), independent of the cytology sample type (smear or cell block). Representative images of three different cases of PD-L1 expression from both types of samples are shown in Figure 3B.

However, if we only segregate samples into two groups, positive (tumor cells ≥ 1%) and negative, or high (tumor cells ≥ 50%) and negative/low-positive, the strength of agreement was substantial at 88.9% and 81.5%, respectively (Supplementary Tables S1 and S2, respectively).

## 4. Discussion

Numerous studies have explored the efficiency of EBUS-TBNA in NSCLC to evaluate PD-L1 expression status or the genomic phenotype using single-gene testing, rapid techniques (RTPCR, IHC) or direct sequencing [10,24]. However, very few have evaluated prospectively the efficiency of EBUS-TBNA for using NGS [25,26] or combining both NGS and PD-L1, and fewer still have included tissue for the genomic evaluation for comparison (Supplementary Table S3) [27,28]. To our knowledge, we are the first to provide prospective data comparing EBUS-TBNA vs. tissue for a comprehensive genomic evaluation including PD-L1 and genetic phenotype determination by NGS.

Most of the samples obtained from EBUS-TBNA, 92.8% (39/42), yielded sufficient material to successfully proceed with all of the biomarker evaluations, overtaking the 75.8% (22/29) achieved with biopsies. This is significant, as cytology obtained from EBUS-TBNA was the only source of material for diagnosis in 13 cases (30.9%), which highlights the relevance of employing such material for other purposes apart from diagnosis.

The use of rapid on-site evaluation (ROSE) and an increased number of needle passes was used to improve the genomic and diagnostic yield of EBUS-TBNA. Whereas three needle passes are sufficient in the diagnosis of mediastinal disease [29,30], a minimum of four needle passes might be required to get enough material for molecular testing [31]. Indeed, the average number of needle passes in our cohort was six. Therefore, an optimal on-site coordination between the pneumology and pathology departments is essential to ensure the lower limits of adequacy and guarantee the acquisition of sufficient tumor cell content for molecular testing.

Cytologies, particularly those from EBUS-TBNA, have some limitations over biopsies including a lower preservation of tissue architecture and a reduced amount of tumor cell content, especially using smears. However, they can be a rich source of genetic material as they can be preserved without FFPE, avoiding the risk of fading of PD-L1 IHC expression or DNA quality found with samples with an extended storage time [5,32]. Fielding and colleagues investigated the potential of using DNA extracted from diff-1uik cytology smears versus cell blocks from EBUS-TBNA procedure and observed that smears have a better yield and therefore success in triaging samples to sequencing [33].

In our series, the adequacy of EBUS-TBNA cytologies for genetic testing was high, above 90% for both NGS and nCounter, and was in line with other previously published studies (Supplementary Table S3 [24,25,26,27,28,33,34,35,36,37,38,39,40,41,42,43]). Molecular results in cytology fully agreed with that of tissue testing, including some *EGFR*, *BRAF*, and *MET*∆ex14 alterations, endorsing cytologies as an optimal source of material for genetic testing. 

PD-L1 assessment was also feasible on cytologic material and all EBUS-TBNA samples were successfully tested for PD-L1 expression. Although an 88.9% agreement with tumor biopsies was observed when using a positive–negative PD-L1 cutoff point (TPS ≥ 1%), a lower concordance rate of 74.1% was observed when classifying in three categories (negative, low-, and high-positive). Most of the discordant cases presented higher PD-L1 expression levels in biopsies regardless of the type of cytology sample evaluated (smear or cell block samples).

To date, PD-L1 testing on EBUS-TBNA smear samples has not been validated with any of the commercially available platforms. Several studies have compared PD-L1 testing between EBUS-TBNA and other tissue specimens, reporting a diverse range of concordance rates—between 69.8 and 91.3% [35,37,39,40,41,43]. Whereas some studies have shown lower rates of PD-L1 expression in cytology specimens [32,43,44], others have reported a higher expression in metastatic lymph nodes than in primary tumors [37].

We found an 18.5% incidence of false negative cases at the TPS ≥ 50% cutoff. This is in contrast to the results of Smith, et al. [37,40], who did not find any false-negative case either at the TPS ≥ 1% or the TPS ≥ 50% cutoffs, but is in line with Sakata, et al. [41], where at the TPS ≥ 50% PD-L1 cutoff, EBUS misclassified the status of 53% (8/15) of PD-L1-positive tumors with an overall concordance between EBUS-TBNA samples and surgical resection specimens of 82%.

Based on the data mentioned above, it is unclear whether EBUS-TBNA specimens may misclassify the status of PD-L1, especially when the PD-L1 cutoff of TPS ≥ 50% is applied. Spatial and temporal heterogeneity of PD-L1 expression have been described in NSCLC and may be a plausible explanation of PD-L1 status discordance observed between samples across studies [45,46,47], increasing the risk of false negatives in small samples with low tumor cellularity, such as those obtained from EBUS-TBNA. Moreover, stage differences may account for varying levels of PD-L1 expression and could partially explain the limitations of EBUS in this setting. In our series only 50% of patients were in an advanced stage of the disease.

The limitations of our study include the small number of patients used for comparison, but nevertheless these results contribute to the existing data on the concordance between EBUS samples and histological specimens.

## 5. Conclusions

Our results support the reliability and feasibility of EBUS-TBNA cytology specimens as an optimal source of material for NSCLC genotyping, expanding the percentage of patients that can be screened for biomarkers in the approximately one-third of patients without biopsy. Although PD-L1 testing is achievable in the majority of EBUS-TBNA samples, the lower agreement observed in higher PD-L1 IHC scores reinforces the need to further prospectively evaluate the value of PD-L1 testing with EBUS-TBNA.

## Figures and Tables

**Table 1:** Characteristics of study population (N = 42).

	No. (%) or Mean (Range)
Age (years)	67.1 (52–83)
Sex	
Male	31 (73.8)
Female	11 (26.2)
Smoking status	
Current	20 (47.6)
Former	17 (40.5)
Never	5 (11.9)
Staging by CT or PET	
IIIA	4 (9.5)
IIIB	12 (28.5)
IIIC	2 (4.8)
IVA	6 (14.3)
IVB	18 (42.9)
Histological type	
Adenocarcinoma	24 (57.2)
Squamous cell carcinoma	10 (23.8)
Non-otherwise specified	4 (9.5)
Large cell carcinoma	4 (9.5)

Abbreviations: CT, computed tomography; PET, positron emission tomography.

**Table 2:** Characteristics of the sampled regions by EBUS-TBNA procedure.

EBUS Findings	No. (%) or Mean (Range)
Regions sampled	78
Nodes	63 (80.8%)
Mediastinal	
Subcarinal	22 (28.2)
Right lower paratracheal	19 (24.3)
Left lower paratracheal	7 (9.0)
Right higher paratracheal	2 (2.6)
Lobar	
Right interlobar	7 (9.0)
Left interlobar	3 (3.8)
Right hilar	2 (2.6)
Left hilar	1 (1.3)
Peribronchial primary tumor mass	15 (19.2)
Number passes per each node station/site	6 (1–13)
Lymph node characteristics	
Size by EBUS (mm)	13.7 (5.9–32.7)
SUV by PET	16.9 (0.0–48.5)

Abbreviations: EBUS-TBNA, endobronchial ultrasound-guided transbronchial needle aspiration; PET, positron emission tomography; SUV; standard uptake value.

**Table 3:** Concordance of the NGS results between EBUS-TBNA and biopsy samples.

		Biopsy		
		Positive	Negative	Total
EBUS-TBNA	Positive	18 ^Φ^	0	18
	Negative	0	5	5
	Total	18	5	23

^Φ^, 2 samples were identified with the same driver, but different co-mutations identified by NGS. Concordance samples are in bold. Number of observed agreements: 23 (100% of the observations). Number of agreements expected by chance: 15.2 (65.97% of the observations). Abbreviations: EBUS-TBNA, EBUS-TBNA, endobronchial ultrasound-guided transbronchial needle; NGS, next-generation sequencing.

**Table 4:** Comparison of PD-L1 results between paired cytological and biopsy samples using three cutoffs, negative (tumor cells < 1%), low- (TPS1-49%) and high-positive (TPS ≥ 50%) (*N* = 27).

		Biopsy			
		Negative	Low-Positive	High-Positive	Total
EBUS-TBNA	Negative	4	2	1	7
	Low-positive	0	5	4	9
	High-positive	0	0	11	11
	Total	4	7	16	27

Number of observed agreements: 20 (74.1% of the observations). Number of agreements expected by chance: 9.9 (36.6% of the observations). Kappa = 0.591 (95% confidence interval from 0.344 to 0.838). Abbreviations: EBUS-TBNA, endobronchial ultrasound-guided transbronchial needle.

## Data Availability

The data presented in this study are available on request from the corresponding author. The data are not publicly available due to privacy.

## Supplementary Materials

The following are available online at https://www.mdpi.com/article/10.3390/cancers13092084/s1, Figure S1: Techniques performed on cytological and biopsy samples (*N* = 42). Patients with paired samples are colored in yellow; Table S1: Comparison of PD-L1 status between paired cytological and biopsy samples (*N* = 27); Table S2: Comparison of PD-L1 immunostaining between paired EBUS-TBNA specimens and biopsy samples (*N* = 27); Table S3: Characteristics of included studies exploring PD-L1 expression and/or other predictive biomarkers relevant in NSCLC tested by NGS in EBUS-TBNA samples.
