# Supplementary material for: EBUS-TBNA Cytological Samples for Comprehensive Molecular Testing in Non–Small Cell Lung Cancer
Source: Cancers (Basel). 2021 Apr 25;13(9):2084. doi: 10.3390/cancers13092084 (PMC8123471; doi:10.3390/cancers13092084)
Supplement: Supplementary file 1 [file cancers-13-02084-s001.zip › cancers-1167443-supplementary.pdf]

Supplementary Material

EBUS-TBNA Cytological Samples for Comprehensive Molecular Testing in Non–small Cell Lung Cancer

Roberto Martín-Deleon, Cristina Teixido, Carmen M Lucena, Daniel Martinez, Ainhoa Fontana, Roxana Reyes, Mireia García, Nuria Viñolas, Ivan Vollmer, Marcelo Sanchez, Pedro Jares, Francisco M Pérez, Naiara Vega, Elba Marin, Ramón M Marrades, Carlos Agustí, Noemi Reguart.

Supplementary Material and Methods

DNA and Oncomine

Briefly, DNA isolation and Next-Generation Sequencing (NGS) were performed with the *QIAamp DNA FFPE Tissue kit* (Qiagen, Hilden, Germany) and the Oncomine™ Solid Tumour DNA Panel Kit (OST, ThermoFisher Scientific, Waltham, USA), respectively, both according to the manufacturer's instructions and as described previously[1]. Ten ng was used as a template to generate genomic libraries. The Oncomine™ Solid Tumour DNA Panel (ThermoFisher Scientific, Waltham, USA), interrogates somatic mutations (substitutions, insertions, deletions and inversions) on 22 genes, including *EGFR*, *ALK*, *ERBB2*, *ERBB4*, *FGFR1*, *FGFR2*, *FGFR3*, *MET*, *DDR2*, *KRAS*, *PIK3CA*, *BRAF*, *AKT1*, *PTEN*, *NRAS*, *MAP2K1*, *STK11*, *NOTCH1*, *CTNNB1*, *SMAD4*, *FBXW7*, and *TP53*. Amplicons were covered on average to a minimum of 500X. To be considered suitable for NGS testing, tumor cells must comprise at least 20% of cells in the sample and there must be more than 300 tumor cells in total.

RNA and nCounter

RNA from formalin-fixed paraffin-embedded (FFPE) and cytological smear samples was extracted with a high purity FFPE RNA isolation kit (Roche, Meylan, France) following the manufacturer's instructions. The total RNA (10-200ng) was hybridized with a custom-designed mixture of biotinylated capture tags and fluorescently labeled reporter probes (*ALK*, *ROS1*, *RET*, *NTRK1*, *METexΔ14*; Elements Chemistry). The panel was customized to identify *ALK*, *ROS1*, *RET*, and *NTRK1* gene fusions, and *MET*Δex14 [2]. Sample processing, imaging, and counting were performed with nCounter Prep Station and Digital Analyzer automated instruments (NanoString Technologies Inc, Seattle, WA) according to the manufacturer's instructions. Expression levels were collected and normalized with the nSolver analysis software version 2.6 [3, 4]. FFPE samples were considered for analysis if they contained a minimum of 500 cells in total with at least 10% tumor cells.

PD-L1 immunohistochemistry

Immunohistochemistry (IHC) was performed on cytological smears or four-micron deparaffinized, formalin-fixed tissue sections using the PD-L1 IHC 22C3 pharmDx assay (Dako, Glostrup, Denmark; on a Dako Autostainer Link 48) with antigen retrieval and antibody dilutions following manufacturer's recommendations. Human tonsil tissue was used as control. PD-L1 expression in tumor cells was evaluated based on the percentage of PD-L1 positive on the membrane intensity staining pattern. Cases were classified according to the Tumor Proportion Score (TPS) as follows: negative (0 and <1%); low-positive (1–49%); and high-positive (50–100%). A minimum of 100 tumor cells were evaluated per sample and PD-L1 positivity was considered when a membrane stain (partial or complete) of tumor cells ⩾1% was observed[5].

Figure S1. Techniques performed on cytological and biopsy samples (N = 42). Patients with paired samples are colored in yellow.

		P1	P2	P3	P4	P5	P6	P7	P8	P9	P10	P11	P12	P13	P14	P15	P16	P17	P18	P19	P20	P21
EBUS-TBNA	NGS	✓	✓	✓	▦	✓	✓	✓	✓	✓	◎	✓	✓	◎	✓	✓	✓	✓	✓	✓	✓	✓
	nCounter	✓	✓	✓	✓	✓	✓	✓	✓	✓	◎	✓	✓	✓	✓	✓	✓	✓	✓	✓	✓	✓
	IHC	✓	✓	✓	✓	✓	✓	✓	✓	▦	✓	✓	✓	✓	✓	✓	✓	✓	✓	✓	✓	✓
Biopsy	NGS	✓	✓	✓	◎			✓		✓	◎	✓	✓	◎	◎	✓		✓	✓	◎	◎	
	nCounter	◎	✓	✓	◎			✓		✓	◎	✓	✓	✓	◎	✓		✓	✓	◎	◎	
	IHC	✓	✓	✓	◎			✓		✓	✓	✓	✓	✓	✓	✓		✓	✓	✓	✓	

		P22	P23	P24	P25	P26	P27	P28	P29	P30	P31	P32	P33	P34	P35	P36	P37	P38	P39	P40	P41	P42
EBUS-TBNA	NGS	✓	✓	✓	✓	✓	✓	◎	✓	✓	✓	✓	✓	✓	✓	✓	✓	✓	✓	✓	✓	✓
	nCounter	✓	✓	✓	✓	✓	✓	◎	✓	✓	✓	✓	✓	✓	✓	✓	✓	✓	✓	✓	✓	✓
	IHC	✓	✓	✓	✓	✓	✓	✓	✓	✓	✓	✓	✓	✓	✓	✓	✓	✓	✓	✓	✓	✓
Biopsy	NGS	✓		✓		✓	✓					✓	✓	✓	✓	✓	✓	✓	✓		✓	
	nCounter	✓		✓		✓	✓					✓	✓	✓	✓	✓	✓	✓	✓		✓	
	IHC	✓		✓		✓	✓					✓	✓	✓	✓	✓	✓	✓	✓		✓	

✓ Done
▦ No evaluable
(empty) No biopsy
◎ Insufficient material

Abbreviations: Endobronquial ultrasound guided transbronchial needle aspiration (EBUS-TBNA), immunohistochemistry (IHC), next-generation sequencing (NGS), patient (P).

Table S1. Comparison of PD-L1 status between paired cytological and biopsy samples (N = 27).

		Biopsy		Total
		Positive	Negative	
EBUS-TBNA	Positive	**20**	0	20
	Negative	3	**4**	7
	Total	23	4	27

Number of observed agreements: 24 (88.9% of the observations). Number of agreements expected by chance: 18.1 (66.9% of the observations). Kappa = 0.664 (95% confidence interval from 0.326 to 1.000). Abbreviations: EBUS-TBNA, endobronchial ultrasound-guided transbronchial needle.

Table S2. Comparison of PD-L1 immunostaining between paired EBUS-TBNA specimens and biopsy samples (N = 27).

		Biopsy		Total
		TPS≥50%	TPS<50%	
EBUS-TBNA	TPS≥50%	**11**	0	11
	TPS<50%	5	**11**	16
	Total	16	11	27

Number of observed agreements: 22 (81.5% of the observations). Number of agreements expected by chance: 13.0 (48.3% of the observations). Kappa= 0.642 (95% confidence interval from 0.377 to 0.906). Abbreviations: EBUS-TBNA, endobronchial ultrasound-guided transbronchial needle; TPS, tumor proportion score.

Table S3. Characteristics of included studies exploring PD-L1 expression and/or other predictive biomarkers relevant in NSCLC tested by NGS in EBUS-TBNA samples.

Reference	Year	Smear/ Cell Block	N	Techniques	Molecular Tests	Comparison histology vs. EBUS-cytology	Study	Success Rate/ Concordance	Other results / Comments
[6]	2020	Cell Block	40	IHC	PD-L1	No	Retrospective	Success Rate 80% (N=32)	PD-L1 expression in advanced NSCLC
[7]	2020	Cell Block	53	IHC	PD-L1	Yes	Retrospective	Sensitivity 73.3%/Specificity 65.2%/VPN 65.2%/VPP 69.8% Concordance 69.8% cutoff TPS≥1% (N=37)	EBUS-TBNA samples are suitable for PD-L1 testing, especially using cutoff of TPS≥50%, concordance of 79.2% (42 pts)
[8]	2020	Cell Block	189	IHC	PD-L1	No	Retrospective	Success Rate 94.7% (N=179)	EBUS sample are adequate for PD-L1 testing
[9]	2020	Cell Block	120	IHC	PD-L1	Partial (N=18)	Retrospective	Success Rate 92% (N=110) Concordance 78% (N=18)	PD-L1 higher in EBUS than histologic samples; 5/18 discordant cases TPS≥50%
[10]	2019	-	50 (TBNA 27/ Stylet 23)	NGS/FISH/ IHC	NGS/*ALK*/ PD-L1 (Techniques not specified)	No	Retrospective	Success Rate (Stylet Retracted Partially *vs* Completely): NGS 88.9% (N=24)/91.3% (N=21); *ALK* 88.9% N=24)//87% (N=20)/; PD-L1 85.2% (N=23)/ 87% (N=20)	Diagnostic yield comparison of EBUS-TBNA *vs* Stylet removal for molecular testing. PD-L1 cutoff TPS >50 or <50%.
[11]	2019	Cell Block	85	NGS/IHC/ RTPCR/ FISH	*EGFR*/*ALK*/ *ROS1*/ NGS (Lung Corel, 56 genes)	No	Prospective	Success Rate 91% (N=77)	100% concordant results between single gene and NGS testing
[12]	2019	Cell Block	71	IHC	PD-L1	Yes	Retrospective	Concordance : EBUS *vs* TBB 83.8% (N=68) EBUS *vs* Resected Primary 84.6% (N=13) EBUS *vs* Resected Metastases 86.7% (N=15)	Concordance: TPS cutoff 1% PD-L1 TPS higher in resected tumors than in EBUS-TBNA
[13]	2019	Cell Block	265	IHC	PD-L1	Partial (N=34)	Prospective	Success Rate 86.8% (N=230) Concordance 91.3% (N=31)	TPS≥50% higher in EBUS EBUS-FNA 100% agrees with histology samples- same anatomic site (N=16)
[14]	2019	Both	67	NGS	TruSeq Amplicon Cancer Panel 48 gene	No	Prospective	Success Rate: 92.5% Concordance: 73% (N=33)	EBUS-TBNA smears vs EBUS-TBNA cell-blocks Smears are recommended as primary source of DNA for NGS in EBUS-TBNA
[15]	2018	Cell Block	61	IHC	PD-L1	Yes	Retrospective	Concordance: 87% cutoff TPS≥1% (N=53) 82% cutoff TPS≥50% (N=50)	EBUS *vs* Resected Using a cutoff TPS≥50%, EBUS-TBNA specimens may misclassify the status of PD-L1
[16]	2018	Cell Block	398	Sanger or NGS /IHC/ FISH/ RTPCR	*EGFR*/ *KRAS*/ *ALK*/ *BRAF*/ *PI3K* /*HER2* NGS (Sentosa SQ NSCLC, 11 genes)	No	Prospective	Success Rate: 79.4% (all techniques, N=316)	RTPCR test (*EGFR*/*KRAS*) in 43 cases EBUS-FNA provides high-quality material for tumor genotyping

[17]	2018	Cell Block	50	NGS / FISH/ IHC	NGS (GatorSeq 76 genes)/ *ALK*/ PD-L1	No	Retrospective	Success Rate: 82% (all techniques, N=41)	EBUS-TBNA is effective and has a high proportion of satisfactory results for testing PD-L1 expression on tumor cells in addition to NGS and ALK FISH
[18]	2018	Cell Block	115	NGS	NGS (MSK IMPACT, 368 genes)	No	Retrospective	Success Rate: 86.1% (N=99)	EBUS-TBNA reliably provided adequate tissue for hybrid capture NGS
[19]	2018	Cell Block	69	PCR, direct sequencing/ FISH	*EGFR*/*KRAS*/ *ROS1*/*ALK*	No	Prospective	Success Rate: 69.6% (all techniques, N=48)	EBUS-TBNA with a 21-gauge needle is appropriate for the analysis of multiple mutations and the genotyping of lung adenocarcinoma
[20]	2017	Both	54	NGS	OncoScreen (50 genes) OncoPlus (1213 genes) panels	No	Retrospective	Success Rate: OncoScreen, 98.0% (N=49); OncoPlus, 91.4% (N=32)	Success rate of NGS testing of EBUS samples utilizing 22- & 25-gauge needles. The size of the needle does not seem to affect the success rate of NGS tests
[21]	2017	Cell Block	97	IHC	PD-L1	Yes	Prospective	Concordance: All r=>0.48, P<.087 EBUS *vs* resected primary r=0.75, P=.086, N=6, EBUS *vs* lymph node r=0.93, P=.02, N=5); EBUS & TBB r=0.75, P<0.001, N=16; TBB & primary tumor r=0.52, P<0.001, N=41; lymph node & primary tumor r=0.48, P<0.001,N=47	EBUS-TBNA is a promising method to evaluate PD-L1 expression. No cutoff of PD-L1 was used

Abbreviations: EBUS guided fine-needle aspiration (EBUS FNA), endobronquial ultrasound-guided transbronchial needle aspiration (EBUS-TBNA), immunohistochemistry (IHC), fluorescent in situ hybridization (FISH), next-generation sequencing (NGS), non-small cell lung cancer (NSCLC), reverse transcription polymerase chain reaction (RTPCR), transbronchial biopsy (TBB), tumor proportion score (TPS), positive predictive value (VPP), negative predictive value (VPN).

References

1. Castillo, P.; Marginet, M.; Jares, P.; García, M.; Gonzalvo, E.; Arance, A.; García, A.; Alos, L.; Teixidó, C. Implementation of an NGS panel for clinical practice in paraffin-embedded tissue samples from locally advanced and metastatic melanoma patients. Explor. Target Antitumor Ther. 2020, 1, 101–108, doi:10.37349/etat.2020.00006
2. Marin, E.; Teixido, C.; Carmona-Rocha, E.; Reyes, R.; Arcocha, A.; Viñolas, N.; Rodríguez-Mues, M.; Cabrera, C.; Sánchez, M.; Vollmer, I.; et al. Usefulness of Two Independent DNA and RNA Tissue-Based Multiplex Assays for the Routine Care of Advanced NSCLC Patients. *Cancers* **2020**, *12*, 1124, doi:10.3390/cancers12051124.
3. Reguart, N.; Teixidó, C.; Giménez-Capitán, A.; Paré, L.; Galván, P.; Viteri, S.; Rodríguez, S.; Peg, V.; Aldeguer, E.; Viñolas, N.; et al. Prat A. Identification of ALK, ROS1, and RET fusions by a multiplexed mRNA-based assay in formalin-fixed, paraffin-embedded samples from advanced non-small-cell lung cancer patients. Clin. Chem. 2017, 63, 751–760.
4. Reguart, N.; Teixido, C.; Gimenez-Capitan, A.; Vilariño, N.; Arcocha, A.; Jares, P.; Castillo, S.; Bernal, X.; Muñoz, S.; Palmero, R.; et al. P1.01-075 Simultaneous Multiplex Profiling of Gene Fusions, METe14 Mutations and Immune Genes in Advanced NSCLC by NCounter Technology. *J. Thorac. Oncol.* **2017**, *12*, S1923, doi:10.1016/j.jtho.2017.09.729.
5. Teixidó, C.; Vilariño, N.; Reyes, R.; Reguart, N. PD-L1 expression testing in non-small cell lung cancer. *Ther. Adv. Med Oncol.* **2018**, *10*, doi:10.1177/1758835918763493.
6. Mineura, K.; Hamaji, M.; Yoshizawa, A.; Nakajima, N.; Kayawake, H.; Tanaka, S.; Yamada, Y.; Yutaka, Y.; Nakajima, D.; Ohsumi, A.; et al. Diagnostic yield of endobronchial ultrasound-guided transbronchial needle aspiration to assess tumor-programmed cell death ligand-1 expression in mediastinal lymph nodes metastasized from non-small cell lung cancer. *Surg. Today* **2020**, *50*, 1049–1055, doi:10.1007/s00595-020-01989-6.
7. Jug, R.; Giovacchini, C.X.; Liu, B.; Green, C.L.; Clarke, J.M.; Mahmood, K.; Pavlisko, E.N. EBUS-FNA cytologic-histologic correlation of PD-L1 immunohistochemistry in non–small cell lung cancer. *J. Am. Soc. Cytopathol.* **2020**, *9*, 485–93, doi:10.1016/j.jasc.2020.04.003.
8. Perrotta, F.; Nankivell, M.; Adizie, J.B.; Maqsood, U.; Elshafi, M.; Jafri, S.; Lerner, A.D.; Woolhouse, I.; Munavvar, M.; Evison, M.; et al. Endobronchial Ultrasound-Guided Transbronchial Needle Aspiration for PD-L1 Testing in Non-small Cell Lung Cancer. Chest 2020, 158, 1230–1239.
9. Smith, A.; Wang, H.; Zerbo, A.; Beaudoin, S.; Ofiara, L.; Fiset, P.-O.; Benedetti, A.; Gonzalez, A.V. Programmed Death Ligand 1 Testing of Endobronchial Ultrasound–guided Transbronchial Needle Aspiration Samples Acquired for the Diagnosis and Staging of Non–Small Cell Lung Cancer. J. Bronchol. Interv. Pulmonol. 2020, 27, 50–57, doi:10.1097/lbr.0000000000000623.
10. Fernandez-Bussy, S.; Biswas, A.; Labarca, G.; Jantz, M.A.; Mehta, H.J. Comparison of Endobronchial Ultrasound–guided Transbronchial Needle Aspiration With Stylet Retracted Partially Versus Completely for Molecular Testing. J. Bronchol. Interv. Pulmonol. 2019, 26, 222–224, doi:10.1097/lbr.0000000000000596.
11. Xie, F.; Zheng, X.; Mao, X.; Zhao, R.; Ye, J.; Zhang, Y.; Sun, J. Next-Generation Sequencing for Genotyping of Endobronchial Ultrasound-Guided Transbronchial Needle Aspiration Samples in Lung Cancer. *Ann. Thorac. Surg.* **2019**, *108*, 219–226, doi:10.1016/j.athoracsur.2019.02.010.
12. Yoshimura, K.; Inoue, Y.; Karayama, M.; Tsuchiya, K.; Mori, K.; Suzuki, Y.; Iwashita, Y.; Kahyo, T.; Kawase, A.; Tanahashi, M.; et al. Heterogeneity analysis of PD-L1 expression and copy number status in EBUS-TBNA biopsy specimens of non-small cell lung cancer: Comparative assessment of primary and metastatic sites. Lung Cancer 2019, 134, 202–209, doi:10.1016/j.lungcan.2019.06.002.
13. Wang, G.; Ionescu, D.N.; Lee, C.-H.; Hiruki, T.; Myers, R.; Shaipanich, T.; Lam, S.; Melosky, B.; Zhou, C. PD-L1 testing on the EBUS-FNA cytology specimens of non-small cell lung cancer. Lung Cancer 2019, 136, 1–5, doi:10.1016/j.lungcan.2019.07.033.
14. Fielding, D.; Dalley, A.J.; Bashirzadeh, F.; Singh, M.; Nandakumar, L.; Reed, A.E.M.; Black, D.; Kazakoff, S.; Pearson, J.V.; Nones, K.; et al. Diff-Quik Cytology Smears from Endobronchial Ultrasound Transbronchial Needle Aspiration Lymph Node Specimens as a Source of DNA for Next-Generation Sequencing Instead of Cell Blocks. *Respir.* **2019**, *97*, 525–539, doi:10.1159/000495661.
15. Sakata, K.K.; Midthun, D.E.; Mullon, J.J.; Kern, R.M.; Nelson, D.R.; Edell, E.S.; Schiavo, D.N.; Jett, J.R.; Aubry, M.C. Comparison of Programmed Death Ligand-1 Immunohistochemical Staining Between Endobronchial Ultrasound Transbronchial Needle Aspiration and Resected Lung Cancer Specimens. Chest 2018, 154, 827–837, doi:10.1016/j.chest.2018.07.017.
16. Ghigna, M.-R.; Crutu, A.; Florea, V.; Feuillet-Soummer, S.; Baldeyrou, P.; Adam, J.; Lacroix, L.; Besse, B.; Mercier, O.; Fadel, E.; et al. Endobronchial ultrasound-guided fine-needle aspiration for pulmonary carcinomas genotyping: experience with 398 cases including rapid EGFR/KRAS analysis in 43 cases. *J. Thorac. Dis.* **2018**, *10*, 4653–4658, doi:10.21037/jtd.2018.06.157.
17. Biswas, A.; Leon, M.E.; Drew, P.; Fernandez-Bussy, S.; Furtado, L.V.; Jantz, M.A.; Mehta, H.J. Clinical performance of endobronchial ultrasound-guided transbronchial needle aspiration for assessing programmed death ligand-1 expression in nonsmall cell lung cancer. Diagn. Cytopathol. 2018, 46, 378–383, doi:10.1002/dc.23900.
18. Turner, S.R.; Buonocore, D.; Desmeules, P.; Rekhtman, N.; Dogan, S.; Lin, O.; Arcila, M.E.; Jones, D.R.; Huang, J. Feasibility of endobronchial ultrasound transbronchial needle aspiration for massively parallel next-generation sequencing in thoracic cancer patients. Lung Cancer 2018, 119, 85–90.
19. Raad, S.; Hanna, N.; Jalal, S.; Bendaly, E.; Zhang, C.; Nuguru, S.; Oueini, H.; Diab, K. Endobronchial Ultrasound-Guided Transbronchial Needle Aspiration Use for Subclassification and Genotyping of Lung Non-Small-Cell Carcinoma. *South. Med J.* **2018**, *111*, 484–488, doi:10.14423/smj.0000000000000846.

20. Stoy, S.P.; Segal, J.P.; Mueller, J.; Furtado, L.V.; Vokes, E.E.; Patel, J.D.; Murgu, S. Feasibility of Endobronchial Ultrasound-guided Transbronchial Needle Aspiration Cytology Specimens for Next Generation Sequencing in Non–small-cell Lung Cancer. Clin. Lung Cancer 2018, 19, 230–238.e2, doi:10.1016/j.cllc.2017.11.010.
21. Sakakibara, R.; Inamura, K.; Tambo, Y.; Ninomiya, H.; Kitazono, S.; Yanagitani, N.; Horiike, A.; Ohyanagi, F.; Matsuura, Y.; Nakao, M.; et al. EBUS-TBNA as a Promising Method for the Evaluation of Tumor PD-L1 Expression in Lung Cancer. Clin. Lung Cancer 2017, 18, 527–534.e1, doi:10.1016/j.cllc.2016.12.002.
